# The Medico-Legal Aspect of Hypnotism

**Published:** 1887-01

**Authors:** 


THE MEDICO-LEGAL ASPECT OF HYPNOTISM.—M. Liégeois, Professor of Law of the Faculty of Nancy, calls attention to the possibility of crimes being committed by hypnotized individuals at the suggestion of those who have put them in this condition, and argues very justly that in such a case the punishment should fall on the author of the criminal suggestion rather than upon the irresponsible agent.—*Medical Record.*

